# A Review of Asynchronous Byzantine Consensus Protocols

**DOI:** 10.3390/s24247927

**Published:** 2024-12-11

**Authors:** Zhenyan Ji, Xiao Zhang, Jianghao Hu, Yuan Lu, Jiqiang Liu

**Affiliations:** 1 School of Cyberspace Science and Technology, Beijing Jiaotong University, Beijing 100044, China; zhyji@bjtu.edu.cn (Z.J.); jqliu@bjtu.edu.cn (J.L.); 2 School of Software Engineering, Beijing Jiaotong University, Beijing 100044, China; 23121591@bjtu.edu.cn (X.Z.); hujianghao503@163.com (J.H.); 3 Institute of Software, Chinese Academy of Sciences, Beijing 100190, China

**Keywords:** blockchain, consensus protocols, asynchronous network model, Byzantine fault tolerance

## Abstract

Blockchain technology can be used in the IoT to ensure the data privacy collected by sensors. In blockchain systems, consensus mechanisms are a key technology for maintaining data consistency and correctness. Among the various consensus protocols, asynchronous Byzantine consensus protocols offer strong robustness as they do not rely on any network timing assumptions during design. As a result, these protocols have become a research hotspot in the field of blockchain. Based on different structural design approaches, asynchronous Byzantine consensus protocols can be divided into two categories: protocols based on the DAG structure and protocols based on the ACS structure. The paper describes their principles and summarizes the related research works. The advantages and disadvantages of the protocols are also compared and analyzed. At the end of the paper, future research directions are identified.

## 1. Introduction

Blockchain is essentially a chain structure that links blocks in chronological order, characterized by decentralization, traceability, and immutability [1,2,3,4]. In 2008, Satoshi Nakamoto first introduced Bitcoin [5], bringing attention to the underlying blockchain technology. In recent years, blockchain technology has rapidly evolved and is now widely applied in fields such as finance [6], supply chain management [7], and the Internet of Things (IoT) [8,9,10,11,12]. The application of blockchain technology in IoT can effectively ensure the data privacy collected by sensors. For example, in the Internet of Medical Things (IoMT) [8], sensors collect vital health information, such as biometric signals, health conditions, etc. Most of the information is sensitive and should be protected. Thus, blockchain can be used to keep the health data’s confidentiality, integrity, and immutability.

Consensus mechanisms are crucial for maintaining the decentralization of blockchain distributed ledgers. They ensure the consistency of data across all network nodes, protect against malicious attempts to alter or damage data for self-serving purposes, and guarantee security and trustworthiness. The development of consensus algorithms is a core driving force behind the continuous improvement of blockchain technology.

Consensus protocols have evolved from the original Proof of Work (PoW) [5] to newer protocols such as Proof of Stake (PoS) [13] and Delegated Proof of Stake (DPoS) [14]. The development of asynchronous consensus protocols has also progressed significantly, moving from the Honey Badger of BFT protocols (HB-BFT) [15] to the Dumbo family of protocols, including Dumbo, SpeedingDumbo and Dumbo-NG [16,17,18]. Additionally, there have been advancements in asynchronous protocols based on Directed Acyclic Graph (DAG) structures, notably the reputation-based Hashgraph consensus protocol [19] and DAG-Rider [20]. The emergence of these consensus protocols has broadened the potential for extensive application of blockchain technology.

Compared to traditional consensus protocols, asynchronous Byzantine consensus protocols exhibit high resilience, tolerating arbitrary network delays, while ensuring system security and reliability. Recently, scholars have conducted extensive research on asynchronous Byzantine consensus protocols. Based on different structural design approaches, these protocols can be divided into two categories: those based on directed acyclic graph (DAG) structures and those based on asynchronous common subset (ACS) structures. Furthermore, within the ACS framework, asynchronous Byzantine consensus protocols can be classified into those based on the BKR framework and those based on the CKPS framework [21]. This classification deepens our understanding of how the different design approaches impact Byzantine fault tolerance (BFT) consensus protocols, promoting ongoing development and innovation. We comprehensively review these protocols, clarify the principles, and detail the performance enhancements of notable protocols from each category. Furthermore, we compare the strengths and weaknesses of both categories and propose future directions for optimizing these protocols.

## 2. Consensus Fundamentals

### 2.1. Overview of Consensus

Distributed consensus refers to the process of reaching agreement on a particular state among multiple nodes within a network [22]. Consensus ensures that the data or state displayed externally by multiple nodes is identical under the protocol’s specified conditions. Blockchain consensus is a specific application of distributed consensus.

### 2.2. Network Communication Models and Their Corresponding Consensus Protocols

The design of consensus protocols relies on assumptions about the underlying network. Different assumptions correspond to various network communication models and consensus protocols. Table 1 summarizes the correspondence between consensus protocols and network assumptions.

#### 2.2.1. Synchronous Model

In a synchronous network model, the delay in transmitting messages within the network is less than a certain known value, and the relative speed difference in processing transactions by nodes is also smaller than a certain known value. Thus, in the synchronous model, any node can complete local transactions and transmit messages to other nodes within a defined period. PoW is a common synchronous consensus protocol. The consistency and liveness of synchronous protocols depend on the synchronous assumptions, and such protocols often face significant delays when deployed globally.

#### 2.2.2. Partially Synchronous Model

The partially synchronous model is a middle ground between the synchronous and asynchronous models. This model assumes the existence of a Global Stabilization Time (GST) [28]. Before GST, the system operates in an asynchronous state. After GST, it transitions to a synchronous state. However, GST is unknown, meaning it is impossible to predict when the system will return to a synchronous state. Common partially synchronous consensus protocols include HotStuff [23] and PBFT [24]. The liveness of this type of protocol depends on synchronous assumptions, and its robustness tends to be poor when deployed globally.

#### 2.2.3. Asynchronous Model

In an asynchronous network model, there is no upper bound on the time it takes to transmit information, with only the guarantee that the information will eventually be delivered. The processing speed of nodes is also unknown. The asynchronous model better reflects the way the actual internet functions. Common asynchronous consensus protocols include the Hashgraph, Tusk, Bullshark, Dumbo, Speeding Dumbo, and Dumbo-NG protocols. These protocols do not rely on synchronous assumptions, making them more suitable for global deployment, and without the need to adjust time parameters during implementation.

### 2.3. Byzantine Generals Problem

The Byzantine Generals Problem was first proposed by Lamport and colleagues in 1982 [29]. The main idea describes the way that generals in the Byzantine Empire, who can only communicate via messengers, can reach a consensus on whether to attack or retreat, even when they know that some of the generals may be traitors [30]. Similarly, a blockchain network is essentially a distributed system and, in the presence of malicious nodes, the consensus mechanism ensures that honest nodes in the system reach an agreement on critical information. This issue mirrors the core of the Byzantine Generals Problem.

In a blockchain network, nodes that act maliciously by forging information or responding in bad faith are referred to as Byzantine fault nodes. While nodes that fail to respond due to network issues are called crash fault nodes. Consensus protocols can be categorized into crash fault tolerance (CFT) protocols and Byzantine fault tolerance (BFT) protocols based on their fault tolerance capabilities [31]. CFT protocols ensure the reliability of a distributed system in the event of node crashes, with Paxos [32] and Raft [33] being representative examples. BFT protocols, on the other hand, ensure the system’s reliability even when nodes exhibit arbitrary faults, providing the number of faulty nodes remains below a certain threshold. Representative protocols in this category include PoW, PoS, DPoS, and PBFT.

### 2.4. A Brief Literature Review of Partially Synchronous Consensus Protocols

Partially synchronous Byzantine consensus algorithms are also a key focus of academic research. Therefore, we have provided a brief literature review on this topic. The Practical Byzantine Fault Tolerance (PBFT) algorithm, proposed by Castro et al. [24], was the first practical Byzantine fault tolerance algorithm. However, the PBFT algorithm has issues with complex view-change logic and unpredictable delays during the view-change process, meaning the system cannot provide external services. Gueta et al. [34] proposed the SBFT algorithm to address the scalability challenges of Byzantine consensus algorithms. SBFT supports over 200 nodes participating in consensus and achieves throughput twice that of PBFT. Yin et al. [23] introduced the HotStuff consensus algorithm, which integrates the process of leader rotation into the consensus flow. This design achieves linear view-change characteristics, addressing the high cost of view changes in PBFT. Although partially synchronous Byzantine consensus mechanisms have reached practical performance levels in production environments, they still cannot resolve stagnation issues in asynchronous network environments. As a result, their use in complex network conditions remains limited.

### 2.5. Summary

With the increasing severity of network and node attacks, only asynchronous Byzantine consensus protocols among Byzantine fault tolerance protocols can offer ideal security in adverse internet environments. Specifically, synchronous Byzantine consensus protocols lose consistency and liveness in asynchronous networks, and semi-synchronous Byzantine consensus protocols lose liveness under such conditions. Both exhibit limited robustness and fail to ensure security against internet attacks. Therefore, we aim to conduct a detailed review to gain a comprehensive understanding of asynchronous Byzantine consensus protocols.

## 3. Asynchronous Byzantine Consensus Protocols and Their Classification

Asynchronous Byzantine consensus protocols are highly resilient and designed without relying on any network timing assumptions. Regardless of the types of errors occurring in system nodes, the system’s reliability is guaranteed providing the number of faulty nodes remains below a certain threshold. With the relaxation of network assumptions and increasingly malicious node behaviors, only asynchronous Byzantine consensus protocols exhibit ideal security and greater robustness in adverse internet environments. In real-world internet environments, asynchronous Byzantine consensus protocols offer superior security compared to traditional consensus protocols, making research in this area highly significant.

Based on different structural design approaches, asynchronous Byzantine consensus protocols can be divided into two categories: those based on directed acyclic graph (DAG) structures and those based on asynchronous common subset (ACS) structures. Both types of asynchronous Byzantine consensus protocols avoid the FLP impossibility [35].

DAG-based asynchronous Byzantine consensus protocols ensure eventual consistency by weakening the guarantee of process consistency through the use of a DAG structure [36]. The system cannot ensure that all nodes hold a consistent state view at any given moment, but over time, all honest nodes will eventually achieve state consistency, converging to a consensus state. This approach fundamentally changes the traditional serial working mode of blockchains, improving the speed of transaction confirmation and block generation [37]. It is particularly suited for scenarios requiring high throughput and scalability. Common protocols in this category include Hashgraph, DAG-Rider, Tusk, and Bullshark.

ACS-based asynchronous Byzantine consensus protocols ensure that all honest nodes can reach consensus without any time restrictions, making them ideal for scenarios with strict requirements for asynchronous consensus. ACS-based asynchronous Byzantine consensus protocols can be further divided into two categories [21]: the Ben-Or, Kelmer, and Rabin (BKR) framework [38] and the Cachin, Kursawe, Petzold, and Shoup (CKPS) framework [39]. The BKR framework’s ACS structure is based on a reliable broadcast (RBC) and an asynchronous binary agreement (ABA), with potential for achieving information-theoretic and post-quantum security. Common protocols in this category include HB-BFT, BEAT, and DispersedLedger. The CKPS framework’s ACS structure is based on broadcast and multi-valued validated Byzantine agreement (MVBA), achieving only computational security. Common protocols in this category include Dumbo and Speeding Dumbo. To address the performance issue in the BKR framework, where some ABA instances cannot be executed in parallel, scholars have proposed PACE, which allows ABA instances to be executed fully in parallel [40]. PACE is based on an RBC and a reproposable asynchronous binary agreement (RABA), which allows replicas to change their decisions and vote twice. However, in the ACS structure of PACE, the time complexity remains at O(log*n*), while the FIN framework [41] achieves constant-time execution with only a constant number of RABA instances.

## 4. Asynchronous Byzantine Consensus Protocols

Representative protocols of DAG-based asynchronous Byzantine consensus include Hashgraph, DAG-Rider, Tusk, and Bullshark. In contrast, representative protocols of ACS-based asynchronous Byzantine consensus include the Honey Badger protocol (BKR), DispersedLedger (BKR), Dumbo (CKPS), and Speeding Dumbo (CKPS). The principles of these protocols are explained in the following sections.

### 4.1. DAG-Based Asynchronous Byzantine Consensus Protocols

#### 4.1.1. Hashgraph

Hashgraph [25] combines DAG and PBFT to effectively improve efficiency and security by ensuring eventual consistency. The Hashgraph algorithm operates based on the “gossip about gossip” and “virtual voting” mechanisms, allowing the protocol to function efficiently in a fully asynchronous environment. It guarantees the correctness of consensus results through a strong visibility mechanism.

**Figure 1:**
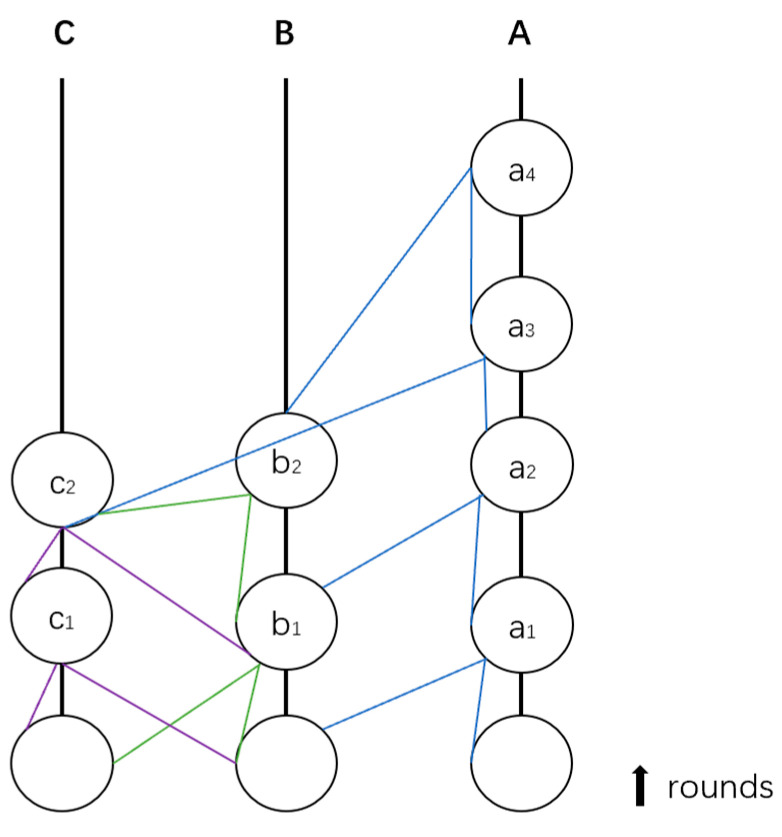
The structure of Hashgraph.

Hashgraph consensus reaches agreement on the global order of events. As shown in Figure 1, A, B, and C represent consensus nodes, and a_1_–a_4_, b_1_–b_2_ and c_1_–c_2_ represent events. The lines between events represent broadcasting. Each node randomly selects a target node to broadcast its latest event. The entire algorithm can be divided into several key steps: First, event creation and propagation take place, where each node propagates events using the gossip protocol, and these events are connected through hash links to form a hashgraph. Next is the round division process, where an event is assigned to the next round if it can observe enough witness events from a given round. Following this is the fame determination process, in which a series of virtual votes decide whether each witness event is “famous”. Finally, the global ordering process occurs where, after confirming the fame of each witness event, the global order of all events is calculated.

#### 4.1.2. DAG-Rider

**Figure 2:**
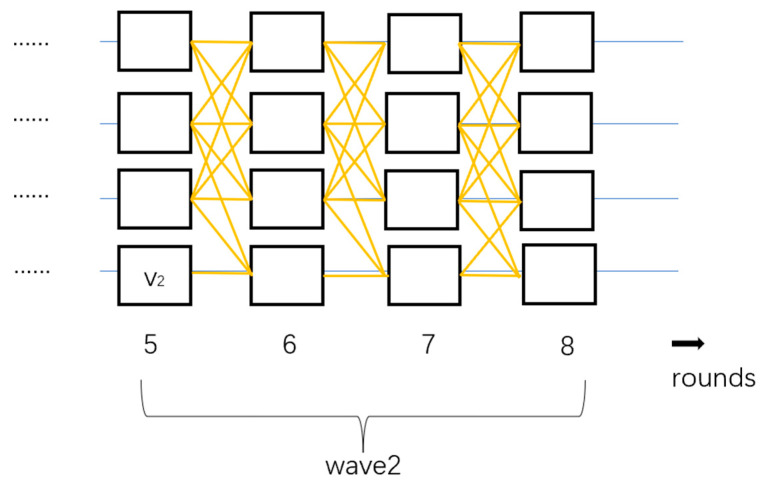
The structure of DAG-Rider. The four horizontal lines represent four different validation nodes, each rectangle represents the consensus messages, and v2 indicates that the fourth node was selected as the leader in the fifth round of wave2.

The DAG-Rider [20] protocol operates through a two-layer structure, ensuring Byzantine fault tolerance while achieving efficient message ordering and decision-making. The communication layer is responsible for broadcasting and receiving proposals and voting messages through reliable broadcast, constructing the messages into a DAG. In the zero-communication overhead ordering layer, each node parses the local DAG and uses randomized methods to order proposals locally. DAG-Rider interprets the DAG as a wave protocol, where a randomly chosen leader vertex is submitted in each wave. Once the leader’s order is determined, all blocks associated with it are sequentially processed. The process is divided into waves consisting of four consecutive rounds, with a leader randomly selected from the first round in the final round of each wave, and attempts are made to commit the chosen vertex as shown in Figure 2.

#### 4.1.3. Tusk

**Figure 3:**
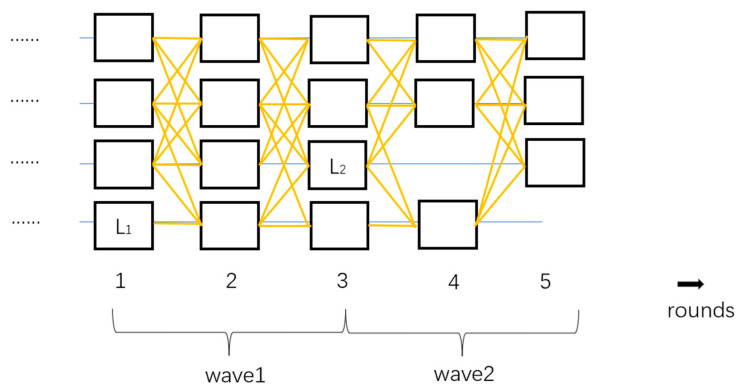
The structure of Tusk.

Tusk [26] improves the commit rules based on DAG-Rider, thus enhancing efficiency. Each wave consists of three rounds, with the last round of one wave serving as the first round of the next wave. In the first round, each validator node generates a block. In the second round, nodes vote on the proposals from other validators. In the third round, a leader is randomly selected from the first round. If f + 1 vertices reference the leader in the second round, the leader is committed; otherwise, the process moves directly to the next wave. As shown in Figure 3, L_1_ is the leader of the first wave, and L_2_ is the leader of the second wave.

#### 4.1.4. Bullshark

**Figure 4:**
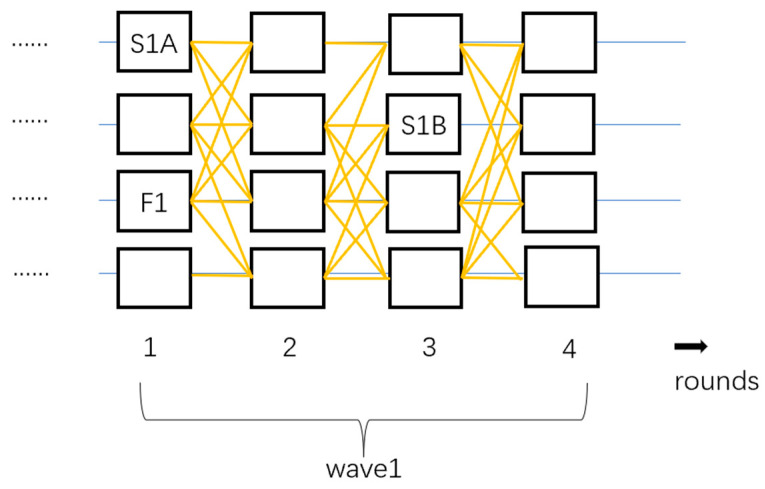
The structure of Bullshark.

Bullshark [27] further reduces latency compared to Tusk. Its asynchronous protocol version includes four rounds per wave, with three leaders being elected. In the first round, a fallback leader is randomly chosen, while both the first and third rounds predefine a steady-state leader. As shown in Figure 4, F1 is the fallback leader, while S1A and S1B are the steady-state leaders.

#### 4.1.5. Summary

The approach of these protocols is to separate the network communication layer from the consensus logic. From DAG-Rider to the Tusk and Bullshark protocols, the continuous optimization of commit rules has reduced latency and improved consensus efficiency.

### 4.2. Asynchronous Byzantine Consensus Protocols Based on ACS Structure

#### 4.2.1. Honey Badger of BFT Protocols (BKR)

**Figure 5:**
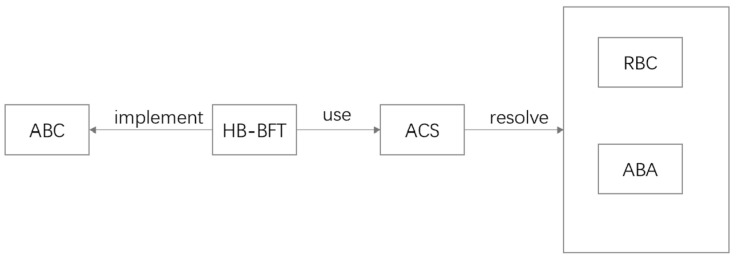
The structure of Hashgraph.

The Honey Badger of BFT Protocols [15] (HB-BFT) is a Byzantine fault tolerance distributed consensus protocol that ensures consistency between nodes in a distributed system. It allows the system to maintain security and liveness even in the presence of Byzantine faulty nodes. The HB-BFT protocol adopts a modular approach to solve the atomic broadcast (ABC) problem in Byzantine environments, which involves ensuring that nodes receive the same messages in the same order in an asynchronous and Byzantine setting. This protocol addresses the ABC problem using an asynchronous common subset (ACS). The ACS can be further decomposed into a reliable broadcast protocol (RBC) and an asynchronous binary agreement (ABA), as shown in Figure 5.

**Figure 6:**
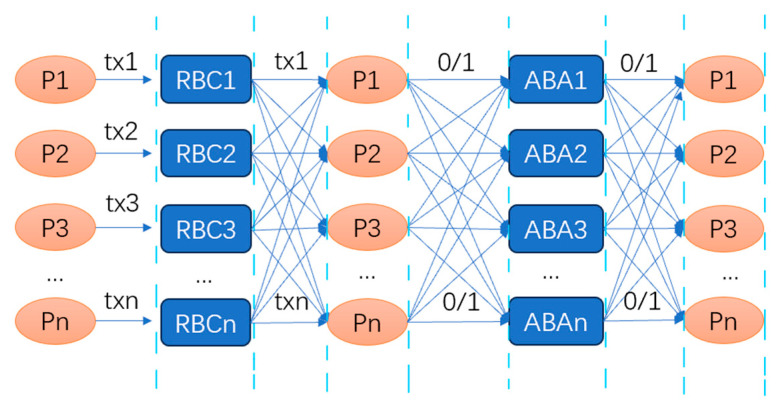
ACS structure in HB-BFT. Tx1-txn represents proposals put forward by nodes, and the lines indicate broadcasting.

The ACS structure is depicted in Figure 6.

The goal of an RBC is to ensure that each node receives proposal messages from all other nodes, forming the foundation for message transmission in the HB-BFT protocol. The nodes verify the validity of messages through mutual signature verification and acknowledgment mechanisms, and they use retransmission to handle message loss caused by network unreliability. This ensures that the subsequent protocol modules in HB-BFT can work in coordination based on correct inputs, ultimately achieving consensus.

The ABA protocol is another core submodule within the ACS framework. The purpose of asynchronous binary agreement (ABA) is to allow all nodes to reach consensus on either 0 or 1 in an asynchronous environment. In HB-BFT, each node executes a binary consensus for every other node’s RBC success, with N ABA instances being executed in parallel in each round of consensus.

In summary, the entire consensus process relies on the RBC to propagate the proposal messages from the nodes and on the ABA to reach agreement on the content.

#### 4.2.2. DispersedLedger (BKR)

**Figure 7:**
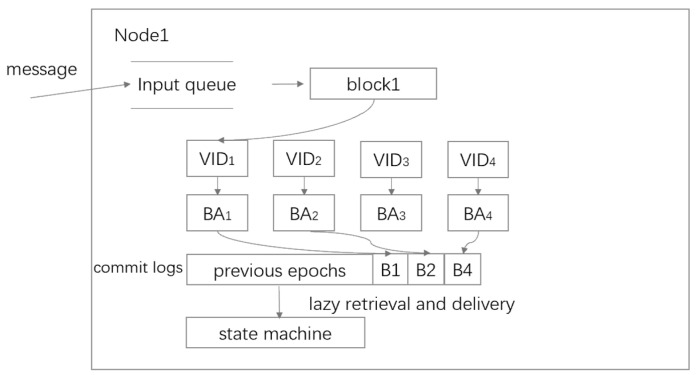
DispersedLedger structure with node 4.

The DispersedLedger [42] protocol builds upon improvements made to the HB-BFT protocol. Unlike HB-BFT, nodes in the DispersedLedger protocol do not broadcast their proposed blocks. Instead, they use AVID-M (referred to as VID) to disperse the proposed blocks across the entire cluster, significantly reducing communication costs. DispersedLedger then relies on N instances of binary agreement (BA) to determine which proposed blocks have been successfully dispersed and to commit consensus during the current epoch. Once committed, nodes can retrieve blocks lazily at any time. The structure of the protocol is illustrated in Figure 7. Each VID instance corresponds to a block proposed by a node. Each BA instance is used to reach a consensus on whether a block has been successfully dispersed. B1, B2, and B4 are blocks proposed by nodes, which are dispersed and processed through VID and BA instances.

#### 4.2.3. Dumbo (CKPS)

**Figure 8:**
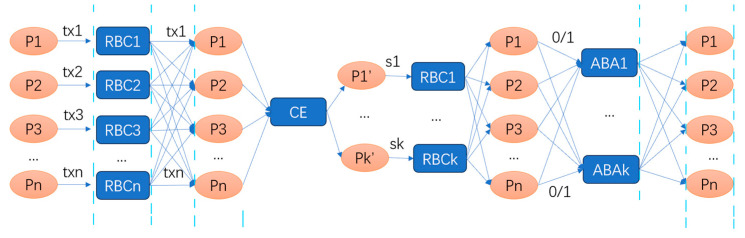
ACS structure of Dumbo1. Tx1-txn represents proposals put forward by nodes, and the lines indicate broadcasting. RBC means reliable broadcast and ABA means asynchronous binary agreement.

In the HB-BFT protocol, the ABA module is the main factor affecting performance due to its high time and communication costs. Therefore, reducing the number of ABA instances in each consensus round is a key approach to improving efficiency. The Dumbo1 protocol [16] reduces the number of ABA instances from n to k by utilizing a committee, transforming the critical issue into how to randomly select a committee of k members while ensuring, with a practical-level probability, that at least one member is honest. The ACS structure of this protocol is shown in Figure 8.

This protocol can be divided into five stages. In the first stage, each node broadcasts its input transactions via an RBC. In the second stage, a committee of k members is randomly selected. In the third stage, the committee members broadcast the indices of the messages they received in the first stage via an RBC. In the fourth stage, each node inputs 1 or 0 into the ABAj instance, depending on whether it has received the corresponding index Sj from node j: it inputs 1 if it has received Sj and 0 otherwise. In the fifth stage, the set of indices with an ABA output of 1 is obtained, and the corresponding value set is sorted and output.

**Figure 9:**
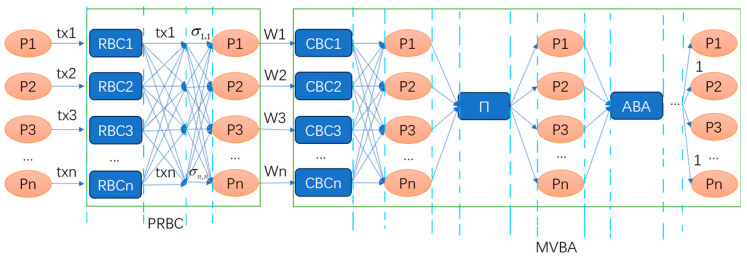
ACS structure of Dumbo2. Tx1-txn represents proposals put forward by nodes, and the lines indicate broadcasting. RBC means reliable broadcast and ABA means asynchronous binary agreement. CBC means Consistent Broadcast.

The Dumbo2 protocol [16] further reduces the number of ABA instances to 2–3. Its ACS structure is shown in Figure 9.

This protocol can be divided into four stages. In the first stage, a reliable broadcast with proof is output. In the second stage, proposals are broadcast using the Consistent Broadcast (CBC) protocol, and each node waits for proposals from other nodes. In the third stage, a random and uniform selection order of node proposals is generated using threshold coin tossing. In the fourth stage, nodes execute an ABA consensus on each proposal in the order determined in the third stage, stopping once a consensus result of 1 is reached. The final consensus result is then output.

Dumbo2 introduces the Provable Reliable Broadcast (PRBC), enhancing the RBC by adding an extra round of threshold signatures to provide a concise proof.

The CBC protocol is one of the core modules of the Multi-Valued Byzantine Agreement (MVBA) module. For example, in a system with four nodes (P0, P1, P2, P3, with P0 as the leader), node P0 sends a CBC_SEND message to all nodes, including itself, to broadcast the original message. Each node then signs the received message using its private key (CBC_ECHO message) and returns it to the leader. The leader continues to receive CBC_ECHO messages from other nodes, verifying each signed message with the public key. Once the number of verified messages exceeds N-f, the leader collects these signatures and broadcasts the collection to all nodes (CBC_FINAL message). Upon receiving this, each node checks if the number of signatures is N-f and verifies each signature with the public key before outputting the original message along with the signature collection.

The MVBA module implements consensus proposals. First, the CBC protocol module broadcasts the proposals, followed by the ABA protocol module reaching consensus on the proposals in the predetermined order. Finally, the consensus proposal is output. In Dumbo2, the PRBC prepares input vectors from a sufficient number of nodes, while the MVBA outputs one of them, reducing the number of ABA instances from n to 2–3.

#### 4.2.4. Speeding Dumbo (CKPS)

**Figure 10:**
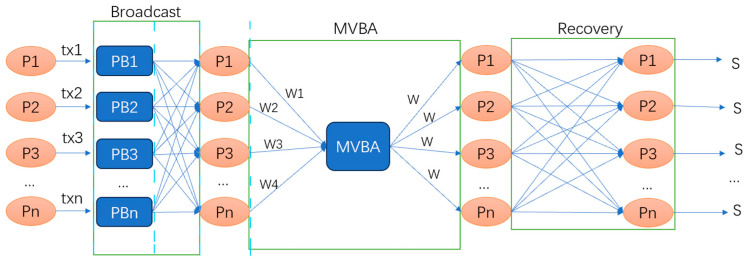
ACS structure of Speeding Dumbo. Tx1-txn represents proposals put forward by nodes, and the lines indicate broadcasting. MVBA means Multi-Valued Byzantine Agreement. PB means provable broadcast.

Speeding Dumbo [17] introduces a new asynchronous BFT structure, which is divided into three stages: broadcast, MVBA, and recovery, as shown in Figure 10.

In the broadcast stage, the sender transmits the transaction to each node, and each node signs the transaction and returns it to the sender. The sender can then output a lock certificate. When the sender generates a valid lock certificate, it guarantees that f + 1 honest nodes have received the same broadcast message.

**Figure 11:**
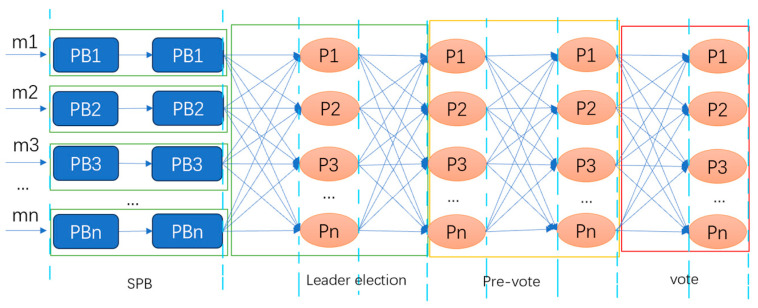
MVBA structure of Speeding Dumbo. M1-mn represents proposals put forward by nodes, and the lines indicate broadcasting.

The protocol introduces a new MVBA protocol, Speeding MVBA, which reduces the number of interaction rounds. In the best-case scenario, it reduces the interaction rounds of the MVBA protocol to six, as shown in Figure 11.

First, Speeding MVBA reduces the number of rounds in the election phase. Second, it reduces the number of PB broadcasts and introduces two voting phases, Pre-Vote and Vote, to address consistency and termination issues. Finally, it introduces an output shortcut, allowing nodes to skip the Pre-Vote and Vote phases under optimistic conditions.

#### 4.2.5. Summary

Asynchronous Byzantine consensus protocols based on the ACS structure can be further divided into two categories: those based on the BKR framework and those based on the CKPS framework. The BKR framework’s DispersedLedger protocol improves upon the HB-BFT protocol, increasing throughput while reducing latency. The CKPS framework’s Speeding Dumbo protocol enhances the Dumbo protocol by using PB broadcast to reduce the complexity of the broadcast phase and to introduce the new Speeding-MVBA.

## 5. Performance Analysis

Asynchronous Byzantine consensus protocols, based on a Directed Acyclic Graph (DAG) structure, transform the single-chain structure into a DAG to enable concurrent writing, offering high throughput and fast processing speeds. These protocols separate network communication from consensus logic. Starting from DAG-Rider and progressing through Tusk and Bullshark protocols, they continually optimize submission rules to reduce latency, as illustrated in Table 2.

Compared to DAG-based blockchain protocols, asynchronous Byzantine consensus protocols, based on the ACS structure, require less memory. Protocols such as Dumbo and DispersedLedger are improvements upon the HB-BFT protocol, while Speeding Dumbo further enhances Dumbo. These advancements have led to improvements in runtime complexity, throughput, and latency, as outlined in Table 2.

Protocols based on the BKR framework, including HB-BFT and DispersedLedger, have the potential to achieve post-quantum security and information-theoretic security by using a universally secure coin protocol. The runtime complexity of these protocols is O(log n). On the other hand, protocols based on the CKPS framework, such as Dumbo and Speeding Dumbo, rely on cryptographic pairing techniques that have not yet been fully validated, and they only achieve computational security. The runtime complexity of these protocols is O(1).

In terms of latency, Dumbo reduces the number of ABA instances compared to HB-BFT. As the system scales, the latency growth rate of Dumbo2 is significantly lower than that of HB-BFT, with a 94% reduction in latency when the system has 64 nodes [16]. Speeding Dumbo introduces a more efficient Speeding-MVBA, reducing the number of execution rounds and decreasing latency by approximately 50% compared to Dumbo [17]. DispersedLedger achieves consensus without downloading complete blocks, reducing data transmission costs and cutting latency by 74% compared to HB-BFT [42].

In terms of throughput, Dumbo2’s new ACS structure reduces the number of ABA instances, resulting in a 320% increase in throughput compared to HB-BFT when the system has 64 nodes [16]. Speeding Dumbo leverages PB broadcast to reduce the complexity of the broadcast phase, significantly lowering communication costs and doubling the throughput compared to Dumbo [17]. DispersedLedger improves throughput by decomposing the consensus process and creating an ordered log. The first step of the consensus process is bandwidth-light, while the second step is bandwidth-intensive, further enhancing throughput. Additionally, creating an ordered log requires less bandwidth than downloading complete blocks, achieving double the throughput compared to HB-BFT [42].

Despite these improvements, both types of protocols still have certain limitations due to their structural designs. Asynchronous Byzantine consensus protocols, based on the DAG structure, consume increasing amounts of memory as the number of nodes grows. For the second type of protocol, performance gradually declines as the number of nodes increases [18].

## 6. Challenges of Asynchronous Byzantine Consensus Protocols

### 6.1. Performance Challenges

While asynchronous consensus protocols are highly robust in adversarial networks, their inherent reliance on randomness increases complexity and slows execution speed. Consequently, the protocols perform poorly compared to deterministic protocols in networks with complex state changes. It has been observed in Bolt-Dumbo Transformer (BDT) [43] that even some of the fastest asynchronous protocols, such as Dumbo and its improved version, Speeding Dumbo, still require approximately ten rounds to complete on average. In contrast, deterministic (partially) synchronous protocols require fewer rounds in synchronous networks, with dual-chain HotStuff completing in just five rounds and PBFT in three rounds.

### 6.2. Technical Challenges

Memory management and garbage collection pose significant challenges for DAG-based asynchronous consensus protocols. If the garbage collection mechanism fails to promptly clean up historical data, memory usage can grow rapidly. Moreover, inappropriate garbage collection may result in the discarding of unconfirmed transactions, thereby impacting both fairness and consistency. Some implementations of ACS-based asynchronous Byzantine consensus protocols rely on threshold encryption, which can increase computational overheads in practical deployments, particularly in resource-constrained environments.

## 7. Future Research Directions

Based on the analysis of the research progress and the strengths and weaknesses of the two types of asynchronous Byzantine consensus protocols, we can foresee several future development directions for these protocols: (a) Researchers can focus on optimizing asynchronous Byzantine consensus protocols by further reducing the memory usage of DAG-based consensus protocols. (b) Asynchronous Byzantine consensus protocols also face scalability challenges. For example, the message complexity of protocols such as Tusk, Bullshark, HB-BFT, and Dumbo is O(n^2^|v| + λn^3^logn). For small networks, the quadratic term (n^2^|v|) is more influential than the cubic term due to its lower growth rate. As network size increases, the cubic term (λn^3^logn) dominates, leading to exponential growth in communication complexity. So, asynchronous Byzantine consensus protocols can be enhanced by improving sharding methods to achieve better scalability. (c) Efforts can also be made to improve the ACS design paradigm by proposing protocols that enable concurrent execution of broadcasting and consensus, thereby overcoming the performance limitations of existing ACS protocols.

## 8. Conclusions

Asynchronous Byzantine consensus protocols can be divided into two categories based on different structural design approaches: DAG-based asynchronous Byzantine consensus protocols and ACS-based asynchronous Byzantine consensus protocols. Furthermore, ACS-based protocols can be categorized into two subtypes: BKR-based and CKPS-based asynchronous Byzantine consensus protocols. This paper has explained the principles of representative protocols from both categories and provided a performance analysis in terms of runtime complexity, throughput, and latency. The advantages and disadvantages of each type were also summarized. The first category achieves high throughput and fast processing by transforming the single-chain structure into a DAG to enable concurrent writes, but it also increases memory usage. The second category of protocols consumes less memory than the first, but they suffer a noticeable decline in performance as the number of nodes increases. Lastly, future research directions for both types of protocols were explored, providing valuable insights for further study.

## Figures and Tables

**Table 1:** Consensus protocols corresponding to different network assumptions.

Classification	Characteristics	Representative Protocols
Synchronous Consensus Protocols	Requires any message in the network to reach all consensus nodes within a known, predetermined time limit.	PoW [5]
Partially Synchronous Consensus Protocols	Any message in the network can reach all consensus nodes within a limited time, but this limit is unknown, and the system eventually assumes synchrony.	HotStuff [23], PBFT [24]
Asynchronous Consensus Protocols	There are no restrictions on the message transmission delay in the network.	HB-BFT [15], Dumbo [16], Hashgraph [25], Tusk [26], Bullshark [27]

**Table 2:** Performance analysis of the introduced protocols.

Classification Based on Structural Design	Protocol	Runtime Complexity	Latency	Throughput	Advantages	Disadvantages
DAG-Based Asynchronous Byzantine Consensus Protocols	Hashgraph [17]	O(exp(n))	low	high	High throughput, fast processing speed	High memory usage
	DAG-Rider [19]	O(1)	high	low		
	Tusk [31]	+∞	medium	medium		
	Bullshark [33]	O(1)/+∞	low	high		
ACS-Based Asynchronous Byzantine Consensus Protocols	HB-BFT [13] (BKR)	O(log n)	high	low	Requires less memory compared to DAG-based protocols	Decreased performance as the number of nodes increases
	DispersedLedger [22] (BKR)	O(log n)	medium	medium		
	Dumbo [14] (CKPS)	O(log k)/O(1)	medium	medium		
	Speeding Dumbo [15] (CKPS)	O(1)	low	high		
